# Sensitivity of imatinib-resistant T315I BCR-ABL CML to a synergistic combination of ponatinib and forskolin treatment

**DOI:** 10.1007/s13277-016-5179-7

**Published:** 2016-07-21

**Authors:** Derrick M. Oaxaca, Sun Ah Yang-Reid, Jeremy A. Ross, Georgialina Rodriguez, Joan G. Staniswalis, Robert A. Kirken

**Affiliations:** 1 Department of Biological Sciences, The University of Texas at El Paso, 500 W. University Ave, El Paso, TX 79968 USA; 2 Department of Mathematical Sciences and Border Biomedical Research Center, The University of Texas at El Paso, 500 W. University Ave, El Paso, TX 79968 USA

**Keywords:** Chronic myeloid leukemia, BCR-ABL, Tyrosine kinase inhibitor, Imatinib, Ponatinib, Forskolin

## Abstract

Tyrosine kinase inhibitors (TKIs) have dramatically improved the life expectancy of patients suffering from chronic myeloid leukemia (CML); however, patients will eventually develop resistance to TKI therapy or adverse side effects due to secondary off-target mechanisms associated with TKIs. CML patients exhibiting TKI resistance are at greater risk of developing an aggressive and drug-insensitive disease. Drug-resistant CML typically arises in response to spontaneous mutations within the drug binding sites of the targeted oncoproteins. To better understand the mechanism of drug resistance in TKI-resistant CML patients, the BCR-ABL transformed cell line KCL22 was grown with increasing concentrations of imatinib for a period of 6 weeks. Subsequently, a drug-resistant derivative of the parental KCL22 cell line harboring the T315I gatekeeper mutation was isolated and investigated for TKI drug sensitivity via multi-agent drug screens. A synergistic combination of ponatinib- and forskolin-reduced cell viability was identified in this clinically relevant imatinib-resistant CML cell line, which also proved efficacious in other CML cell lines. In summary, this study provides new insight into the biological underpinnings of BCR-ABL-driven CML and potential rationale for investigating novel treatment strategies for patients with T315I CML.

## Introduction

Chronic myeloid leukemia (CML) is characterized by the presence of the Philadelphia (Ph) chromosome, a chromosomal abnormality arising from a translocation between the long arms of chromosome 9 and chromosome 22 [1]. CML is a neoplasm that originates from the hematopoietic lineage that is categorized by three distinct phases. The chronic phase (CP) is the most treatable and frequently diagnosed stage. The accelerated phase (AP) and blast phase (BP) are the least commonly diagnosed stages and represent end stage of the disease [2]. The Ph chromosome produces a constitutively active protein tyrosine kinase, BCR-ABL. Over the past 15 years, BCR-ABL has served as a hallmark for the identification of CML and has been extensively studied as a key target in the development of chemotherapies, such as imatinib.

Imatinib is an ATP-binding antagonist that interacts with the P-loop/ATP binding site of the BCR-ABL protein. Such binding renders BCR-ABL incapable of activating downstream targets involved in pro-survival signaling pathways such as Janus kinase (JAK), signal transducer and activator of transcription (STAT), SRC family kinase (SFK), phosphatidylinositol 3-kinase (PI3K), protein kinase B (AKT), mitogen-activated protein kinase (MAPK), and RAS signaling pathways [3]. Mortality rates of patients with CML have been significantly reduced with the introduction of imatinib as a therapeutic agent. In a trial of 1503 patients treated with imatinib and monitored for more than 10 years, the resulting progression-free survival rate was 82 % [4]. Importantly, patients within this study also displayed an 84 % overall survival rate with little to no adverse side effects associated with imatinib therapy [4]. Despite having high levels of success in treating CML with imatinib, a significant percentage of CML patients go on to develop drug toxicities or drug resistance resulting in an increased risk of progressing towards the BP stage of disease.

While the underlying mechanisms by which imatinib sensitivity are lost are not entirely understood, it has been reported that 90-point mutations within the BCR-ABL protein comprise the majority of imatinib-resistant CML cases [2]. Recently, second and third generation tyrosine kinase inhibitors (TKIs), such as dasatinib and nilotinib, have been approved to treat resistant forms of CML and have exhibited marked success [5]. Dasatinib can be used as a front-line treatment because of its ability to target SFK signaling pathways downstream of BCR-ABL. However, somatic mutations in BCR-ABL render the protein insensitive to nilotinib or dasatinib treatment. The third-generation TKI, bosutinib, has displayed limited therapeutic potential for treating CML patients expressing somatic mutations found within the BCR-ABL kinase domain, specifically the T315I mutation [6].

Ponatinib, a third-generation TKI, was specifically designed to inhibit BCR-ABL-positive CML cells containing the T315I mutation. Ponatinib is a unique TKI which does not require a hydrogen bond to the native threonine 315 residue required by imatinib, nilotinib, and dasatinib to associate with the BCR-ABL ATP-binding pocket [7]. Phase I clinical trials of ponatinib in refractory Ph chromosome-positive leukemias demonstrated high efficacy against multiple resistant forms including those harboring the T315I mutation [8]. Ponatinib has also been demonstrated to be effective against the T315I TKI-resistant CML cells; however, serious adverse side effects such as blood clots and narrowing of blood vessels were reported [9–11]. Patients receiving TKI therapy are at increased risk of developing hypertension, arterial/venous thromboembolic events, cerebral ischemia, or myocardial infarction due to secondary off-target effects of TKIs. Moreover, CML patients with underlying conditions were found to be more prone to develop adverse reactions such as pulmonary hypertension, peripheral arterial occlusive disease, and venous and arterial vascular occlusive events, if undergoing therapy with second- and third-generation TKIs [12].

Given the limited number of treatment options available for drug-resistant CML patients, new avenues of TKI utilization must be explored in order to reduce adverse side effects associated with treatment and increase patient survival. As such, the current study sought to investigate TKI resistance and identify novel drug combinations with biological and clinical rationale for the treatment of T315I positive CML. The results provided herein suggest that a two-compound combination of ponatinib and forskolin, a natural chemical produced in the roots of the plant *Coleus forskohlii*, works in a synergistic fashion to inhibit drug-resistant CML.

## Results

### Generation and characterization of an imatinib-resistant CML cell line harboring the BCR-ABL T315I mutation

**Fig. 1:**
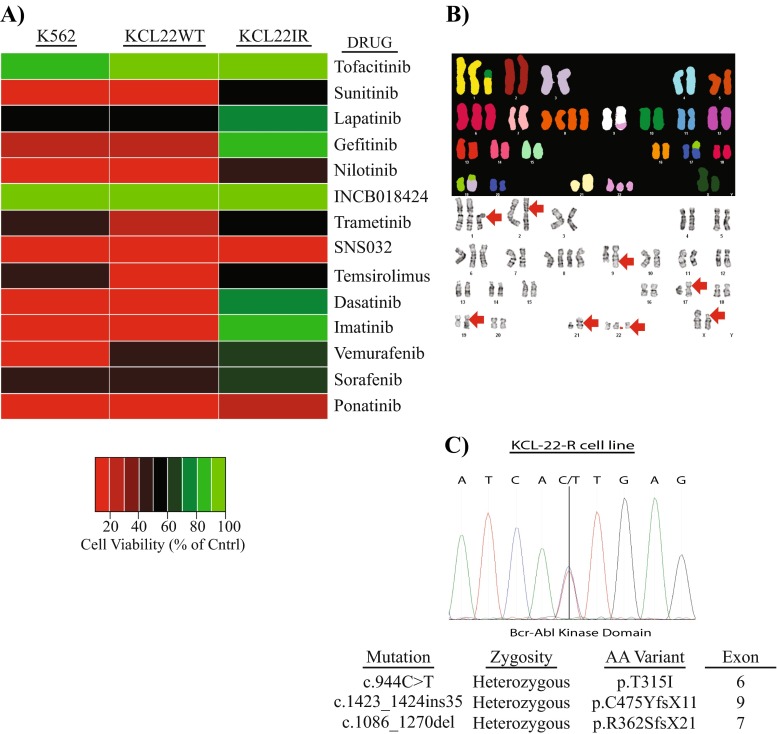
KCL22-IR cells contain the T315I BCR-ABL mutation and demonstrate resistance towards multiple FDA-approved small molecule kinase inhibitors. **a** Heat map representation of cell viability (% of DMSO, Cntrl) in response to reported Cmax values of FDA-approved compounds at 72 h in K562, KCL22-WT, and KCL22-IR cell lines, *n* = 3. **b** Molecular cytogenetic karyotyping analysis of KCL22-IR cell line, SKY type (*top panel*) and G-banded karyotyping (*bottom panel*). Composite karyotype of KCL22-IR is as follows: 50~54,X,del(X)(p11.4p22.2),+der(1;10)(q10;p10),dup(2)(p13p21),+6,+8,+8,t(9;22)(q34;q11.2), der(17;19)(q10;q10),+19,der(19)t(3;19)(q25;q13.1),i(21)(q10),+der(22)t(9;22)[cp17],55,idem,+6,+8[2],51,idem,+6,ad(6)(q13),der(18)t(5;18)(p13;p11.2). **(c)** Sequencing chromatograph of KCL22-IR DNA highlighting the C > T substitution at nucleotide position 944 on Exon 6 of the BCR-ABL kinase domain. The *table* lists a brief description of mutations identified during DNA sequencing

Studies were conducted utilizing the human KCL22 cell line, originally established from the pleural effusion of a 32-year-old female exhibiting Ph-positive CML during BP stage of disease [13]. This cell line has been extensively used as a model to investigate one of the most aggressive stages of CML. Here, KCL22 cells were treated with increasing concentrations of imatinib for 6 weeks as described in the “Materials and Methods” section to generate an imatinib-resistant CML, KCL22-IR, and cell line. KCL22-IR cells were assessed for cell viability in response to multiple FDA-approved kinase inhibitors, chromosomal abnormalities, and mutations within the BCR-ABL coding sequences (Fig. 1). Parental KCL22 (KCL22-WT) and KCL22-IR cells were screened against 14 FDA-approved kinase inhibitors at Cmax-derived dose points for 72 h. Similarly, the unrelated but well-studied human CML cell line, K562, was assessed using the Cmax inhibitory drug concentrations. Following 72-h treatment, cell viability was measured by MTS assay (Fig. 1a). The results indicate that KCL22-IR cells are insensitive to treatment with the majority of the kinase inhibitors used in the screen. Both K562 and KCL22 parental cells displayed sensitivity towards imatinib, dasatinib, nilotinib, and ponatinib as seen by cell viability levels below 20 %. KCL22-IR cells displayed a limited response against both BCR-ABL- and non-BCR-ABL-selective inhibitors such as sunitinib, nilotinib, trametinib, temsirolimus, SNS032, and ponatinib, which were shown to decrease viability by 50 % or more (Fig. 1a).

BCR-ABL^T315I^-selective TKI, ponatinib, was capable of inhibiting all three cell lines tested. KCL22-IR cells demonstrated slightly higher cell viability levels in response to individual treatments with the compounds tested, suggesting an increased resistance to multiple kinase inhibitors.

Karyotyping analysis was performed on KCL22-IR cells to further characterize this CML drug-resistant cell line (Fig. 1b). The results demonstrated an extensive abnormal karyotype consisting of multiple structural and numerical aberrations including the *t*(9;22) which was present with trisomy 6 and tetrasomy 8. Karyotyping analysis also revealed the presence of the Ph-positive chromosome as well as an additional chromosome 22. With the exception of the specific novel KCL22-IR (50~54,X,del(X)(p11.4p22.2),dup(2)(p13p21),der(19)*t*(3;19)-(q25;q13.1), chromosomes identified by karyotyping analysis of the KCL22-IR cell line are in agreement with prior karyotyping of a previously described imatinib-resistant KCL22 cell line [14].

RT-PCR amplification followed by DNA sequencing of the ABL kinase domain covering amino acids 236–486 was carried out to identify novel somatic mutations within the BCR-ABL kinase domain of KCL22-IR cells (Fig. 1c). Mutation of threonine at position 315 to isoleucine (T315I) within the BCR-ABL kinase domain was confirmed in approximately an equal amount to the normal allele. Additionally, two minor variants were also detected. Variant one, c.1087_1088delGA, displayed a frameshift mutation beginning at aspartate 363 and continued for 18 codons resulting in a truncated protein. The clinical significance of this mutation and whether it is responsible for imatinib resistance is unknown. Variant two, a 35-base pair insertion occurring at the junction of exon 8 and exon 9, was also detected and caused a premature truncation of the protein within the BCR-ABL kinase domain. Notably, two mutations, c.1423_1424ins35 and c.1086_1270del, were present and amino acid variations are shown to be p.C475YfsX11 and p.R362SfsX21, respectively. Amino acid variant p.C475YfsX11 has been identified in patients known to have resistance towards multiple kinase inhibitors of BCR-ABL [15, 16]. However, the amino acid variant R362SfsX21 is novel to the KCL22-IR cell line and has yet to be identified and investigated within the general population.

**Fig. 2:**
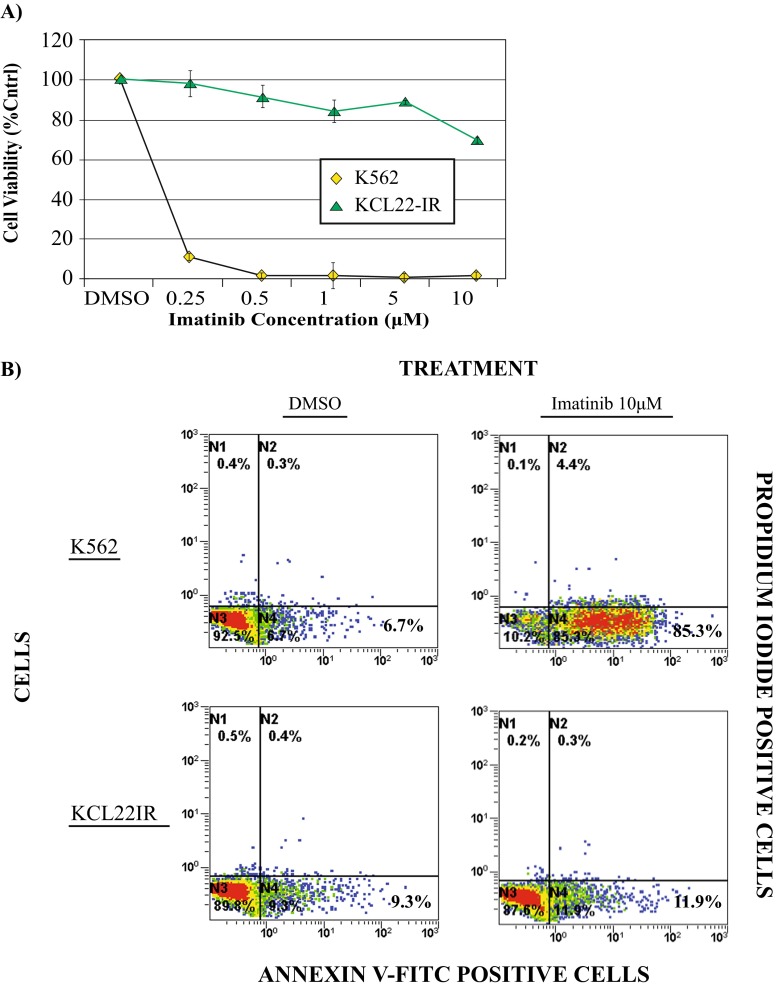
Increased resistance towards high concentrations of imatinib is observed within KCL22-IR cell line. **a** MTS assay showing comparison of cell viability in K562 and KCL22-IR cells treated with vehicle (DMSO) or escalating doses of imatinib for 48 h. *Error bars* represent standard deviation, *n* = 3. **b** FACS analysis of cells treated with vehicle (DMSO) or 10 μM imatinib followed by staining with propidium iodide and Annexin V-FITC. K562 and KCL22-IR cells treated for 48 h as indicated. Annexin V-FITC (*horizontal axis*) and propidium iodide (*vertical axis*) staining was performed to assess apoptosis levels for each treatment, *n* = 3

Lastly, in comparison to the K562 cell line, which displayed 85 % cell apoptosis, the KCL22-IR cell line displayed limited change in cell viability levels (Fig. 2a) or apoptotic response (Fig. 2b), following 48-h treatment with 10 μM imatinib. Such findings are significant as the concentration of imatinib is nearly ten times the approximated clinically attainable value [17].

### Forskolin and celastrol inhibit cell viability within the T315I harboring KCL22-IR cell line

The KCL22-IR cell line was screened against a panel of natural compounds, including celastrol and forskolin. Celastrol has been previously identified to be a selective inhibitor of heat shock protein 90 (Hsp90), which can lead to the down regulation of multiple tyrosine kinases including BCR-ABL [18]. Hsp90, a chaperone of BCR-ABL and other major cell-signaling molecules, has remained an attractive target for designing molecules capable of inhibiting multiple forms of cancer [19]. Recently, celastrol has been reported to induce apoptosis and deplete BCR-ABL protein expression levels within imatinib-resistant CML cells harboring the T315I mutation [18]. Celastrol treatment (1 μM) of KCL22-IR cells showed a ~50 % loss of cell viability when treated for 72-h (data not shown). This concentration is more than 100-fold the Cmax values reported in toxicology reports for Sprague-Dawley rats treated with intravenous and oral administration of pure celastrol [20]. Moreover, an IC_50_ value was attained at 72 h using the adenylate cyclase activator, forskolin, at a concentration of 100 μM. Importantly, this dose of forskolin is 50-fold less than the reported LD_50_ concentrations tested in rats to be tolerable [21]. Forskolin has been demonstrated to impact BCR-ABL signaling by activating protein phosphatase 2A (PP2A) [22]. PP2A is a serine/threonine protein phosphatase that is important in regulating many cellular functions and has been shown to be repressed in several cancer types. Previous studies indicate BCR-ABL kinase activation induced the expression of the PP2A inhibitor, SET [22]. These studies suggest that the effects of BCR-ABL activation are reversible with forskolin treatment.

### Synergistic inhibitory effect of ponatinib and forskolin treatment on cell viability of CML cell lines

**Fig. 3:**
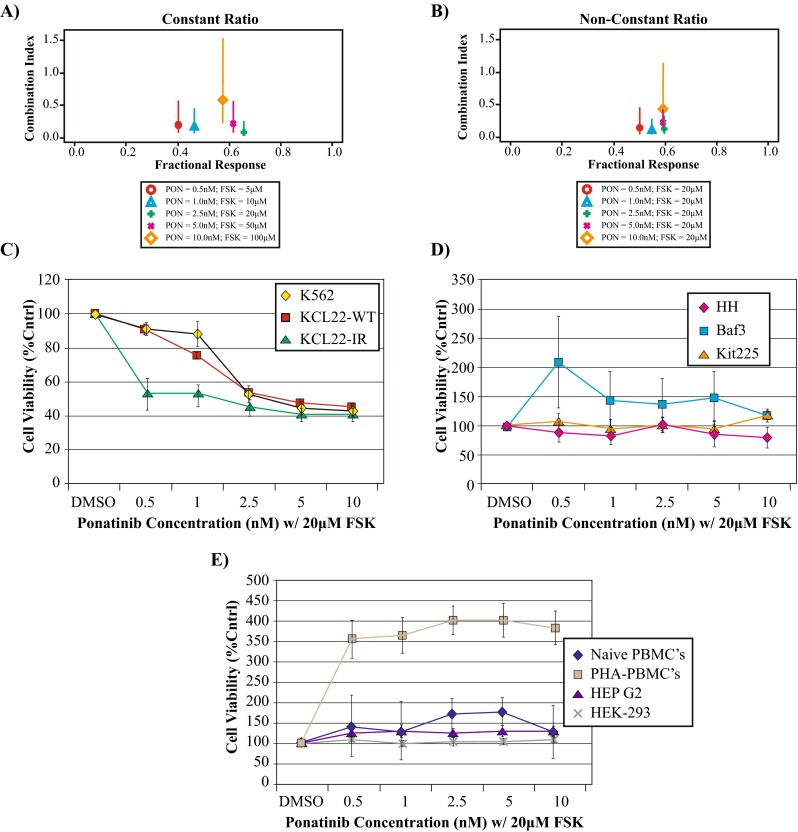
Ponatinib and forskolin combinational treatment shows synergy in KCL22-IR cells and selectivity for CML cell lines. **a** Constant-ratio (1:10,000) combination index plot demonstrating synergistic activity between ponatinib and forskolin treatment by varying ponatinib between 0.5 and 10 nM and forskolin from 5 to 10 μM. The interaction index based on the Loewe Additivity model was used. Values <1, =1, or >1 indicate synergy, additive effect, or antagonism, respectively. **b** Non-constant-ratio combination index plot highlighting 20 μM forskolin maintains synergy with ponatinib at concentrations between 0.5 and 10 nM. **c** CML cell lines K562, KCL22-WT, and KCL22-IR were treated with increasing concentrations of ponatinib, as indicated, in combination with 20 μM forskolin, *n* = 3. **d** Non-BCR-Abl-expressing leukemic cell lines including HH, Baf3, and Kit225 cells were treated with combinational therapy of increasing ponatinib concentrations, as indicated, plus 20 μM forskolin, *n* = 3. **e** Naïve PBMCs, PHA-activated PBMCs, or non-lymphoid tissue cells lines HEP G2 and HEK-293 treated with increasing concentrations of ponatinib, as indicated, plus 20 μM forskolin, *n* = 3

Since the KCL22-IR cell line displayed sensitivity to single-agent treatments with ponatinib and forskolin, combination efficacy with both drugs was investigated. Utilizing the interaction index based on the Loewe Additivity model, a highly synergistic combination of ponatinib and forskolin was identified by varying the doses as indicated in Fig. 3. Utilizing a non-constant-ratio combination, keeping forskolin concentration set at 20 μM, CI values were obtained for five dose points, ranging from 0.5 to 10 nM ponatinib. Results from this combination resulted in CI values ranging from 0.309 to 0.688 (Fig. 3b). The highest synergy was observed in KCL22-IR cells treated with a combination of 2.5 nM ponatinib and 20 μM forskolin (Fig. 3a, b). These findings are important given that the IC_50_ values of individual treatments are 20 nM ponatinib and 100 μM forskolin in KCL22-IR cells.

To evaluate the synergistic effect of ponatinib and forskolin treatment (abbreviated PF), parental KCL22 and K562 cells were employed. PF treatment displayed synergistic activity against these CML cell lines; however, synergistic activity remained prominent within the KCL22-IR cell line (Fig. 3c). To ensure that PF treatment specifically targeted BCR-ABL-positive cells, combinational treatment was also tested against three non-myeloid, BCR-ABL-negative, leukemia cell lines. Cells tested included the IL-3-dependent murine pro-B cell line, Baf3, the IL-2-dependent human T cell line, Kit225, and the human T cell lymphoma cell line, HH. The results indicated that PF treatment did not impact the cell viability of these three BCR-ABL-deficient leukemia cell lines (Fig. 3d). Lastly, PF treatment was tested against human primary naive and PHA-stimulated PBMCs to investigate the off-target effects of treatment. Similar combinational treatment was tested against two non-hematopoietic cells lines, HepG2 and HEK293 (Fig. 3e). As shown in Fig. 3e, there was no observable effect on cell viability following combinational treatment suggesting that PF treatment is selective for BCR-ABL-expressing CML cells. Importantly, these results also suggest that PF combinational treatment has limited off-target effects against non-myeloid BCR-ABL-deficient cell types.

### Ponatinib and forskolin combination modulates KCL22-IR cell-signaling activity downstream of BCR-ABL and does not regulate cell viability through an apoptotic mechanism

**Fig. 4:**
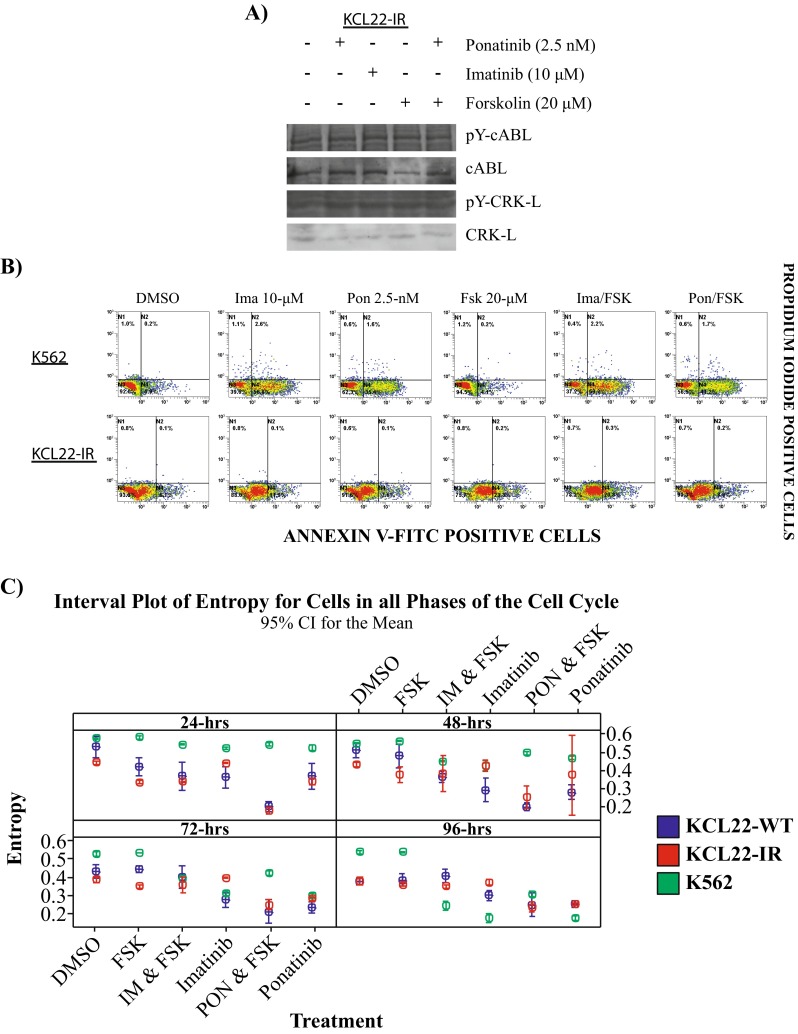
Ponatinib and forskolin treatment regulate cell cycle progression of KCL22-IR cells. **a** KCL22-IR cells were treated with 2.5 nM ponatinib, 10 μM imatinib, and 20 μM forskolin, for 4 h. KCL22-IR cell lysate was separated by SDS-PAGE and Western blotted for phosphotyrosine (pY) cABL, total cABL, pY-CRK-L, and total CRK-L, representative image of *n* = 3. **b** Apoptosis panel of K562 and KCL22-IR cells treated for 48 h with vehicle (DMSO), imatinib, ponatinib, forskolin as indicated, combinational treatments of imatinib (10 μM) plus forskolin (20 μM), or ponatinib (2.5 nM) plus forskolin (20 μM). Annexin V-FITC (*horizontal axis*) and propidium iodide (*vertical axis*) staining was performed to assess apoptosis levels for each treatment, *n* = 3. **c** KCL22-WT, KCL22-IR, and K562 cells were subjected to similar treatment as above for 24, 48, 72, and 96 h. Entropy measurements are shown for each time point, *n* = 3. Analysis for each time point utilizing 0.01 level of significance to control for multiple comparisons at four time points (Bonferroni correction used). *Graphs* display drug response as a measurement of entropy. As percentage of cells shift towards a specific cell cycle phase (amount of cells within G1 phase) entropy decreases, allowing for identification of area of cell cycle arrest (*P* < 0.005)

To investigate the mechanism by which PF treatment induces cell death within the KCL22-IR cell line, cells were treated with ponatinib (2.5 nM), forskolin (20 μM), imatinib (10 μM), or a combination of ponatinib (2.5 nM) with forskolin (20 μM). Following a 4-h treatment, the phosphorylation state of BCR-ABL remained unchanged regardless of treatment conditions within the KCL22-IR cell line (Fig. 4a). In addition, the common BCR-ABL substrate and linker protein CRK-L was shown to have unchanged phosphorylation activity in response to PF treatment (Fig. 4a).

Utilizing the same treatment conditions mentioned above, modulators of apoptosis were analyzed after 48-h treatment with PF in KCL22-IR and K562 cell lines (Fig. 4b). Annexin V/PI staining suggests that KCL22-IR cells displayed no apoptotic mechanism when exposed to these treatment conditions. Additionally, within both cell lines, forskolin (20 μM) alone was not sufficient to induce a significant amount of Annexin V-positive cells. However, the K562 cell line did demonstrate an apoptotic response to imatinib (10 μM) and imatinib (10 μM) in combination with forskolin (20 μM). These results suggest that forskolin alone is not sufficient for inducing apoptosis within BCR-ABL^T315I^-positive CML cells. Interestingly, ponatinib alone (2.5 nM) and PF combinational treatment induced apoptosis specifically in the K562 cell line but had no effect on KCL22-IR cells. To reconcile these findings with previous results showing KCL22-IR cell viability is reduced by PF treatment, we investigated the effects on cell cycle progression. Cell cycle analysis revealed that PF treatment lead to an increase in the percentage of KCL22-IR cells being arrested in the G1 phase in comparison to KCL22 parental and K562 cell lines. Cell cycle arrest within G1 phase was seen in all three cell lines (Fig. 4c).

**Fig. 5:**
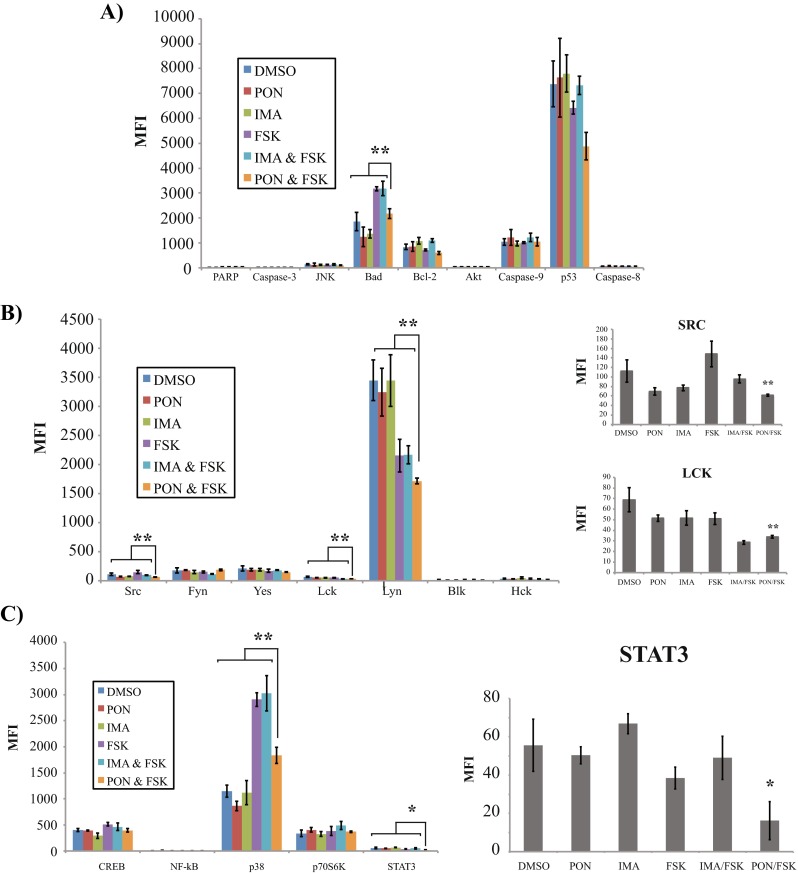
Ponatinib and forskolin treatment of KCL-22-IR cells modulates LYN kinase and STAT3 activation. **a** Multiplex analysis of apoptosis associated proteins in KCL22-IR cells treated with vehicle (DMSO), ponatinib (2.5 nM), imatinib (10 μM), forskolin (20 μM), combinational treatments of imatinib (10 μM) plus forskolin (20 μM), or ponatinib (2.5 nM) plus forskolin (20 μM) for 24 h. *MFI* (median fluorescence intensity) is plotted (*y-axis*) for mean values of three independent tests, *n* = 3. **b** Multiplex analysis of activation status of SFK tyrosine kinase family members in KCL22-IR cells treated as described above, *n* = 3. **c** Multiplex analysis of phosphorylation status of multiple signal transduction proteins after treatment of KCL22-IR cells as described above. *Error bars* indicate standard deviation, *n* = 3; statistical relevance indicated as *p* < 0.01 (*single asterisk*); *p* < 0.001 (*double asterisk*)

To determine whether a 24-h PF treatment had any effect on KCL22-IR signal transduction, the phosphorylation status of multiple targets within various signaling pathways was investigated. After a 24-h PF treatment, apoptosis associated proteins (PARP, Caspase-3, JNK, BCL-2, AKT, Caspase-9, p53, and Caspase-8) showed no significant response to treatment compared to vehicle alone (Fig. 5a). BCL-2 associated death promoter (BAD) showed a significant increase in activation in response to PF treatment in comparison to ponatinib treatment alone (Fig. 5a); however, we note that forskolin treatment alone was sufficient to elevate BAD phosphorylation. As reported earlier, cell viability assays suggest that the concentrations of forskolin used in this study do not significantly alter cell viability within the KCL22-IR cell line.

Based on these findings, SFKs were also analyzed as shown in Fig. 5b, as a possible explanation for reduced cell viability following PF treatment. SRC kinase activity was greatly disrupted by PF treatment when compared to individual treatment of imatinib, ponatinib, forskolin, or the combination of imatinib and forskolin. Indeed, reduced tyrosine phosphorylation in response to PF treatment was observed for SRC, LCK, and LYN kinase activation profiles (Fig. 5b).

Lastly, various substrates that serve important roles in maintaining cell survival, gene transcription, and cell growth were analyzed (Fig. 5c). STAT3 activation was found to be significantly decreased in KCL22-IR cells following PF treatment. In summary, activated forms of LYN-, SRC-, and LCK kinases as well as STAT3 were decreased in response to combinational PF treatment, suggesting that these intermediates are vital players in maintaining cell viability and cell cycle progression within the KCL22-IR cell line.

## Discussion

The imatinib-resistant BCR-ABL T315I mutation in CML has been associated with decreased overall survival rates when compared to non-mutation possessing patients [23]. Ponatinib has demonstrated potent efficacy in drug-resistant forms of CML, including those harboring the T315I mutation; however, ponatinib has proven to be toxic at high concentrations [11, 24]. Overcoming this clinical barrier could entail exploring new strategies to elicit ponatinib efficacy at lower concentrations, such as those described in this study. Combination treatment of ponatinib and forskolin reduced individual IC_50_ values by approximately 40-fold with respect to the observed reduction in viability of the T315I-positive KCL22-IR cell line.

In vitro studies have revealed that IC_50_ levels of ponatinib require doses of approximately 8 nM, while most drug-resistant CML cell lines require 30 nM ponatinib to be effective [9]. The data presented in this study suggest that ponatinib IC_50_ values can be reduced to 0.5 nM when used in combination with forskolin at a concentration of 20 uM. Within the KCL22-IR cell line, treatments with 20 nM ponatinib or 100 μM forskolin individually, were required to obtain any inhibitory effect (data not shown). This synergistic activity may be due to altered BCR-ABL regulation as forskolin is known to induce activation of the phosphatase PP2A, which regulates BCR-ABL activity through activation of SHP1 [22]. It is tempting to speculate that the effects observed may be attributed to two potential mechanisms by which PF treatment inhibits BCR-ABL. First, BCR-ABL inhibition is generated by the direct binding of ponatinib within the BCR-ABL ATP binding site. Second, forskolin induces the activation of PP2A which inhibits BCR-ABL activity directly. It is worth noting that ponatinib possesses unique properties to effectively inhibit the BCR-ABL protein in the presence or absence of the T315I mutation [7].

Based upon the selectivity profiles of each inhibitor assessed in the reported drug screen, several candidate cell-signaling pathways were postulated to be activated. Potential signaling pathways that may be activated within the KCL22-IR cell line include BCR-ABL^T315I^, mammalian target of rapamycin (mTOR), mitogen-activated protein kinase (MEK 1/2), and cyclin-dependent kinase (CDK) 9/2/7 pathways. Various groups have reported that BCR-ABL activity may directly or indirectly modulate multiple signaling pathways including PI3K/AKT and RAS/RAF/MEK/ERK pathway [25]. However, when utilizing TKIs, RTK activity was not critical for cell survival within the KCL22-IR cell line. Furthermore, the results described in this study suggest a novel approach for treating drug resistant CML cases by exploiting an observed synergistic activity between ponatinib in combination with forskolin. To the best of our knowledge, this is the first time ponatinib and forskolin have been investigated for the treatment of drug-resistant CML in vitro, and such a synergistic approach is yet to be tested in patients with T315I-positive CML. The results observed here may be indicative of forskolin’s ability to reactivate the known negative regulator of BCR-ABL, protein phosphatase PP2A [22]. Forskolin has been used globally as a natural herb to treat cardiovascular ailments, skin disorders, hypertension, and other disorders [26]. In addition, forskolin has been clinically evaluated for its therapeutic properties in treating and preventing asthma attacks and has been shown to demonstrate a similar effectiveness when compared to widely used glucocorticoid steroids such as beclomethasone [27]. Combination treatment involving the usage of ponatinib and forskolin may provide a unique opportunity to directly or indirectly target BCR-ABL. This novel treatment strategy may ultimately be a viable option for managing T315I-positive CML cells at lower doses of ponatinib than those currently employed in the clinic.

Current ponatinib treatment has been linked to a significant risk of cardiac toxicity and vascular adverse events (VAEs), such as blood clots, cardiac arrhythmias, congestive heart failure, and myocardial infarction [24]. Indeed, such VAEs have been associated with multiple TKIs, including imatinib [12]. Based upon the results presented herein, further investigation into the new therapeutic strategy in which a combination of ponatinib or other TKIs are used in combination with forskolin is warranted. Forskolin and ponatinib demonstrated a synergistic combination and mechanistic effect on BCR-ABL pathways that may reduce the progression of CML without the risk attributed to VAEs. The results presented in this study identified an imatinib-resistant T315I BCR-ABLCML cell line, KCL-22IR, whose growth could be synergistically inhibited with clinically attainable levels of ponatinib and forskolin, thereby representing a novel therapeutic strategy to treat this drug-resistant type of CML.

## Materials and Methods

### Cell culture and establishment of imatinib-resistant cell line

The following cell lines, KCL22 (parental and IR), K562, HH, and human peripheral blood mononuclear cells (PBMCs) were maintained using complete RPMI-1640 media (HyClone) containing 10 % fetal bovine serum (FBS, Atlanta Biologicals), 2 mM L-glutamine (Corning), 50 mg/ml penicillin-streptomycin (Corning). Kit225 and Baf3 cell lines were maintained in complete RPMI-1640 media supplemented with 100 IU/ml IL-2 or 100 IU/ml IL-3, respectively. HEK293 and HepG2 cells were maintained in DMEM high glucose (HyClone,) containing 10 % FBS, 2 mM L-glutamine and 50 mg/ml penicillin-streptomycin. The imatinib-resistant cell line was developed by maintaining parental KCL22 cells in culture with imatinib concentrations of 500 nM, 750 nM, or 1 μM during weeks 1–2, weeks 3–4, and weeks 4–6, respectively. During this 6-week period, KCL22 cells were passaged at 40 % of the standing population and imatinib concentrations replenished every 3 days. Cell population number was consistently maintained between 5 and 10 million cells per milliliter.

### Drug treatments

Clinically observed peak drug exposure concentrations (Cmax), obtained from literature regarding phase I evaluations, were used for generating appropriate in vitro dose curves for each kinase inhibitor. Cmax values for each kinase inhibitor were as follows: imatinib (4478.0 ng/ml) [17], gefitinib (443.88 ng/ml) [28], dasatinib (46 ng/ml) [29], sunitinib (70.9 ng/ul) [30], erlotinib (1737 ng/ul) [31], lapatinib (2470 ng/ul) [32], nilotinib (1906.27 ng/ml) [33], sorafenib (2248 ng/ul) [34], trametinib (33.4 ng/ml) [35], and vemurafenib (61 μg/ml) [36].

### Cell viability assays

Cells were cultured in complete media (described above) in the absence or presence of the respective Cmax concentration determined for each respective drug dose curve. Cells were seeded at a concentration of 1.0 × 10^4^ cells per well in a 96-well plate. Culture plates were kept in incubation at 37 °C, 5 % CO_2_ for 72-h. After 72-h, CellTiter96 MTS Reagent (Promega) was used according to manufacture instructions. Absorbance was recorded at 490 nm using a 96-well plate reader.

### Cell apoptosis screening

To investigate whether disruption of cellular membrane integrity is involved as a mechanism of cell death, cells were analyzed via flow cytometry. Cells were stained utilizing Annexin V-FITC and PI (Promega). Cells were seeded in 24-well plates at a density of 100,000 cells per well in 2 ml of culture media. Compounds used in the apoptosis screen were imatinib (10 μM), ponatinib (2.5 nM), forskolin (20 μM), imatinib with forskolin (10 and 20 μM, respectively), ponatinib with forskolin (2.5 and 20 μM, respectively), DMSO was used as a negative control, and H_2_O_2_ (10 μM) was used as a positive control. Cells were kept in culture with individual treatments for 48 h, after which were harvested and suspended in cold 1X PBS. Cells were stained according to the manufactures instructions. Briefly, staining consisted of placing cells in 100 μl of cold binding buffer containing a mixture of Annexin V-FITC and PI for 15 min. Cells were analyzed via flow cytometry using a Cytomics FC 500 (Beckman Coulter).

### Luminex analysis of cell-signaling proteins

Protein expression and phosphorylation status were analyzed utilizing a Luminex 100 System (Austin, TX). Milliplex assays were obtained from Millipore, Billerica, MA, USA. Assays used included the following: 9-Plex MultiPathway Panel for phosphorylated ERK/MAP kinase 1/2 (Thr185/Tyr187), Akt (Ser473), STAT3 (Ser727), JNK (Thr183/Tyr185), p70 S6 kinase (Thr412), NF-κB (Ser536), STAT5A/B (Tyr694/699), CREB (Ser133), and p38 (Thr180/Tyr182); 8-Plex Src Family Kinase Panel for phosphorylated Blk (Tyr389), Fgr (Tyr412), Fyn (Tyr420), Hck (Tyr411), Lck (Tyr394), Lyn (Tyr397), Src (Tyr419), and Yes (Tyr421); 3-Plex Apoptosis Panel for activated Caspase3, total GAPDH, and cleaved PARP; and 7-Plex Early Apoptosis Panel for Akt (Ser473), BAD (Ser112), Bcl-2 (Ser70), Active Caspase 8 (Asp384), Active Caspase 9 (Asp315), and JNK (Thr183/Tyr185), p53 (Ser46). Cells were lysed, and luminex plates were prepared and analyzed according to manufacture instructions.

### Solubilization of proteins, electrophoresis, and Western blot analysis

1.0 × 10^6^ cells were treated for 4 h as indicated, lysed, and protein quantified as previously described [37]. Equal concentrations of protein were subjected to SDS-PAGE electrophoresis and protein transferred to PVDF membrane as previously described [37]. Antibodies to cAbl (K-12, Santa Cruz Biotechnology), Crk-L (C-20, Santa Cruz Biotechnology), and phosphotyrosine (4G10, Millipore) were used for Western blotting. All antibodies were used according to manufactures suggested protocols. Western blots were developed with horseradish peroxidase-conjugated goat anti-mouse IgG (heavy plus light chain, KPL) or goat anti-rabbit IgG (heavy plus light chain, KPL) and visualized using enhanced chemiluminescence and x-ray film.

### Drug synergy calculation and statistics

Under the assumption that the dose-effect curves follow Chou-Talalay’s median effect equation [38], Lee and Kong (2009) provide analytic expressions and software for calculating the confidence interval for the estimated interaction index [39]. Drug synergy was determined by use of the CI of Interaction Index software which can be downloaded from http:/biostatistics.mdanderson.org/SoftwareDownload/. The interaction index was calculated using the four doses on the fixed-ray design according to Lee and Kong (2009) and for combination doses according to Lee and Kong (2009). According to the Loewe Additivity model, interaction index (CI) values <1, =1, or >1 represent synergy, additive effect, or antagonism, respectively [38].

### Cell cycle phase entropy calculation

Let *p*_*i*_ denote the proportion of cells found in the *i*th phase of a cell cycle. Then, entropy is

$$ -\sum_i{p}_i \log\ {p}_i $$

where the sum is over all phases of the cell cycle. An entropy of zero indicates that the cells are all in one phase of the cell cycle. Maximum entropy occurs when the proportion of cells in each cell cycle phase is the same.

### G-banding and spectral karyotyping and BCR-ABL kinase domain mutation analysis

KCL22-IR cells were sent to WiCell Research Institute, Madison, WI, USA, for G-banding and spectral karyotyping (SKY) analysis. Qiagen DNeasy Blood & Tissue Kit was used to purify genomic DNA from KCL22-IR cells according to manufactures protocol. DNA concentration was measured using NanoDrop 3000 (Thermo Fisher Scientific), and was verified using 1 % agarose gel (Ultra-pure Agarose with 1× TBE) containing 0.002 % ethidium bromide. KCL22-IR genomic DNA was sent to the Blood Center of Wisconsin, Milwaukee, WI, USA, for identification of DNA variants within the ABL kinase domain by RT-PCR followed by DNA sequence analysis of ABL kinase domain covering amino acids 236–486.
